# Underrepresentation of Hispanics in clinical trials for liver cancer in the United States over the past 20 years

**DOI:** 10.1002/cam4.6814

**Published:** 2023-12-20

**Authors:** Cecilia Monge, Tim F. Greten

**Affiliations:** ^1^ Gastrointestinal Malignancy Section, Thoracic and GI Malignancies Branch, Center for Cancer Research, National Cancer Institute National Institutes of Health Bethesda Maryland USA; ^2^ Liver Cancer Program, Center for Cancer Research, National Cancer Institute National Institutes of Health Bethesda Maryland USA

**Keywords:** cholangiocarcinoma, clinical trials, liver cancer, SEER, diversity

## Abstract

**Importance:**

Accurate representation of racial and ethnic population subgroups in clinical trials is fundamental to ensure universal effectiveness of new therapies as well as to decrease disparities in oncology care.

**Objective:**

To determine whether Hispanic people are underrepresented in Phase I and II clinical trials for liver cancer in the United States.

**Participants:**

A database search was performed in clinicaltrials.gov for interventional liver cancer studies based only in the US with reported results from September 1, 2002, to February 1, 2023. A total of 37 trials with 963 total patients met inclusion criteria and were included for further analysis. Proportion of total patients by race/ethnicity was calculated for non‐Hispanic white, non‐Hispanic black, Asian, Hispanic, and American Indian/Alaska Native subpopulations. The age‐adjusted incidence rates of liver and intrahepatic bile duct were acquired from the National Cancer Institute, Surveillance, Epidemiology, and End Results Program.

**Results:**

Liver cancer incidence rates (per 100,000 people) were highest in Indians/Alaska Native people (18.8 cases) followed by Hispanic people (15.1 cases), then Asian people (12.5 cases), then non‐Hispanic black people (11 cases), and non‐Hispanic white people (7.5 cases). From a total of 91 phase I or II clinical trials for liver cancer in the US, 41% reported race/ethnicity enrollment data; among these, 62.8% of patients were non‐Hispanic White, 15.9% were non‐Hispanic black, 8.8% were Hispanic, 12.7% Asian, and 0.5% American Indian/Alaska Native.

**Conclusions and Relevance:**

Less than half of phase I or II clinical trials for liver cancer in the US in the last 20 years reported race/ethnicity data to clinicaltrials.gov. Compared to the relative incidence rate of liver cancer, non‐Hispanic black people and Hispanic people are underrepresented in these clinical trials.

## Graphical abstract

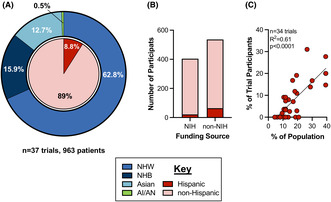

## 1 INTRODUCTION

The American Cancer Society estimates that 1,958,310 new cancer cases and 609,820 cancer deaths are projected to occur in the United States in 2023.^1^ With a rise in novel treatment strategies, the survival rate for cancers of all types has increased from 49% in the mid‐1970s to 68% from 2012 through 2018.^2^ However, access to improvements in oncologic care and survival rates for some cancer types, namely liver cancer, are not equally among all races/ethnicities.^2^, ^3^ While the mortality rates for several types of cancer have improved for the non‐Hispanic white (NHW) population, such rates for other racial/ethnic groups have lagged behind.^4^ Differences in cancer outcomes based on race/ethnicity may be attributed to several factors including socioeconomic (access to care, early detection, risk factors) or genetic factors (tumor biology, treatment efficacy, comorbidities).

In the United States, the two largest ethnic subpopulations, Hispanic people and Non‐Hispanic Black (NHB) people, are growing rapidly, accounting for 19% and 14% of the total population, respectively.^2^ Both populations have been reported to have a relatively high incidence of liver cancer, a leading cause of cancer death worldwide.^5^, ^6^, ^7^ According to the National Cancer Institute, Hispanic people and NHB people make up 23.2% and 11.1% of hepatocellular carcinoma cases, respectively.^2^ Despite the important burden of liver cancer in these populations, they are often underrepresented in clinical trials.^8^, ^9^, ^10^, ^11^ This underrepresentation can result in a lack of precise results including the safety and efficacy of new treatments for these populations, leading to disparities in oncology care. The importance of accurate representation of racial/ethnic subgroups in clinical trials has been highlighted in various studies^8^, ^12^, ^13^, ^14^ and has been supported and mandated by The National Institutes of Health (NIH).^15^, ^16^ Further, clinical trials that lead to FDA approvals may result in the development of treatment guidelines that would presumably be based on the demographics of the participants from such trials. It is therefore critical to ensure that the demographics of the trial participants are representative of the patients who would eventually receive the approved therapy.

Studies have found that Hispanic people and NHB people may respond differently to treatments compared to other racial/ethnic groups.^17^, ^18^ This may be due to biological/genetic differences between different races and ethnicities and/or potentially greater financial burden associated with clinical trial participation for Hispanic and NHB people. US‐born Hispanic people have a higher incidence and lower survival rate compared to foreign‐born Hispanic people for some cancers, and financial toxicity is disproportionately higher in non‐white and Hispanic clinical trial participants.^5^, ^19^ To begin to address these issue, it is critical to understand and improve the enrollment of racial/ethnic groups in clinical trials and to ensure that these trials accurately reflect the population they are intended to serve. This is particularly important in the context of clinical trials for liver cancer, given the high incidence of the disease in Hispanic and NHB populations.

As new therapies continue to be tested in clinical trials for cancer, it is crucial to ensure representation of diverse racial/ethnic populations to test the efficacy and safety of treatment in all subgroups. The aim of this study is to further the understanding of racial/ethnic representation in clinical trials for liver cancer by analyzing enrollment data for phase I and II liver cancer trials conducted within the US in the last 20 years. The results of this study will provide valuable insights into the enrollment of racial/ethnic groups in clinical trials and will inform the development of strategies to increase representation of these groups in clinical trials.

## 2 METHODS

### 2.1 Database for incidence rates

The age‐adjusted incidence rates of liver and intrahepatic bile duct were acquired from the National Cancer Institute, Surveillance, Epidemiology, and End Results Program.^2^ Rates are per 100,000 and are age‐adjusted to the 2000 US Std Population (19 age groups—Census P25‐1130). Incidence data for the category of “liver and intrahepatic bile duct cancer” in the SEER database includes the following cancer sites: liver, intrahepatic bile duct, gallbladder, other biliary, pancreas, retroperitoneum, peritoneum, omentum and mesentery, and other digestive organs.

### 2.2 Selection of clinical trials

A database search was performed in clinicaltrials.gov for phase I and II interventional liver cancer studies based only in the US with reported results from September 1, 2002, to February 1, 2023. Studies were excluded for: having sites outside of the US, being non‐interventional, not reporting results, being phase III or IV, including participants with cancer in other organs, and not including race and ethnicity data, leaving a total of 37 trials for further analysis.

### 2.3 Statistical analysis

The difference in incidence by race and ethnicity (D‐IRE) and the ratio of incidence by race and ethnicity (R‐IRE) were calculated as follows: The liver cancer incidence rates were mathematically “normalized” for each race/ethnicity to the total number of liver cancer cases between 2015 and 2019 to analyze the proportion per 100,000. This was done by dividing the incidence of liver cancer for each of the five races/ethnicities (Hispanic, Asian, NHB, NHW, and AI/AN) by the total number of cancer cases per 100,000 among those five groups to get “% of total cases” in 2015–2019.

Next, the number of clinical trial participants of each race was mathematically “normalized” to the total number of participants to analyze the proportion. This was done by dividing the number of clinical trial participants for each race/ethnicity by the total number of participants to result in “% of total participants for each race/ethnicity.”

Finally, to analyze the D‐IRE, the normalized incidence number was subtracted from the normalized trial enrollment number for each race/ethnicity. The R‐IRE was calculated by dividing the normalized trial enrollment number by the normalized incidence number. D‐IREs below 1 and R‐IREs below 0 indicate under representation in clinical trials based on relative incidence rate. GraphPad Prism^9^ was used for chi‐square and linear regression analysis where appropriate.

## 3 RESULTS

### 3.1 Incidence rates of liver cancer by race/ethnicity

**FIGURE 1:**
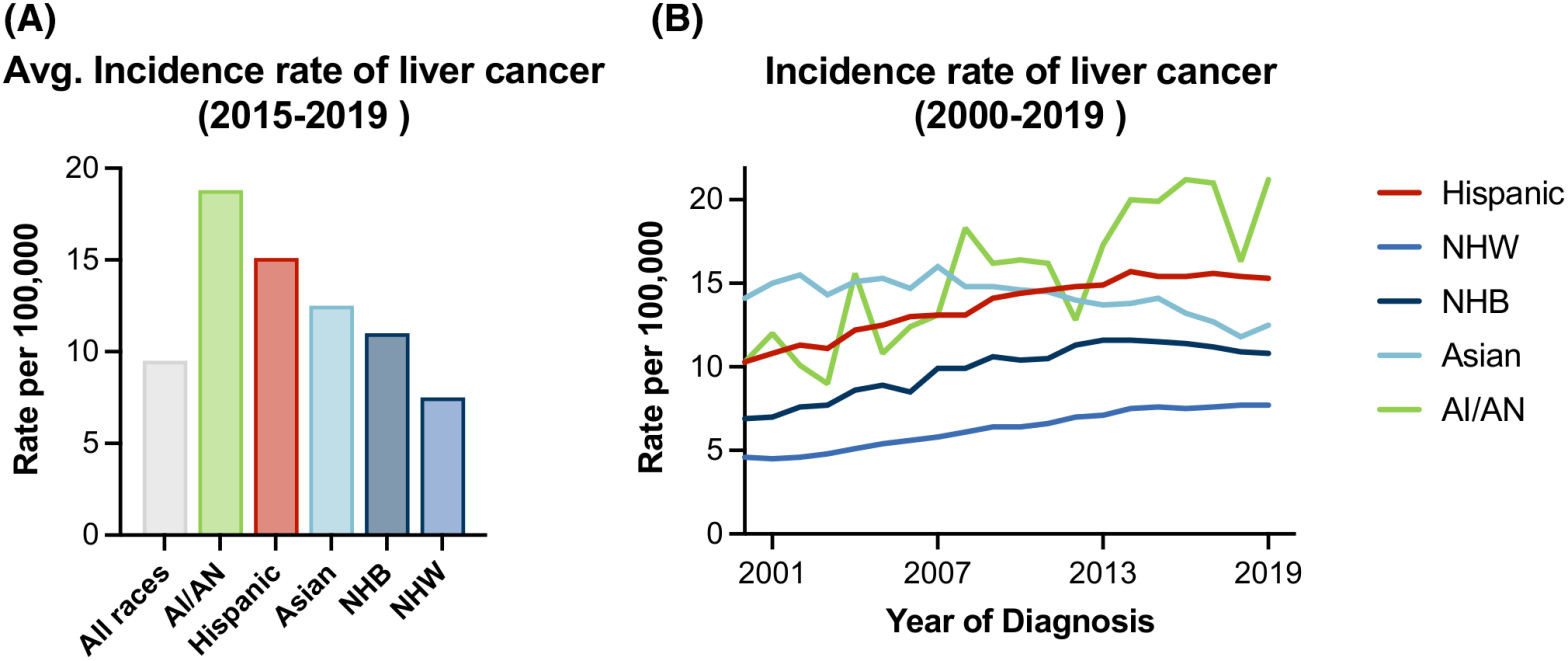
(A) Incidence rates of liver cancer by race/ethnicity. (B) Average incidence rates of liver cancer between 2015 and 2019. Incidence rates of liver cancer per year between 2000 and 2019. AI/AN, American Indian/Alaska Native individuals, NHB, non‐Hispanic black individuals, NHW, non‐Hispanic white individuals. Rates are per 100,000 and are age‐adjusted to the 2000 US Std Population (19 age groups—Census P25‐1130).

Between 2015 and 2019, the average incidence of liver cancer in all races was 9.5 cases per 100,000 people in the US. American Indians/Alaska Natives (AI/AN) had the highest incidence at 18.8 cases per 100,000 people followed by Hispanic people at 15.1, then Asian people at 12.5, then non‐Hispanic black people at 11, and non‐Hispanic white people at 7.5 cases per 100,000 people (Figure 1A). Incidence rates of liver cancer increased over time (between 2000 and 2013) in all groups except in Asian people who instead saw a steady, but relatively high incidence rate. From 2013 to 2019, incidence rates plateaued in Hispanic people, NHB people, and NHW people, decreased in Asian people, and increased in AI/AN people (Figure 1B).

### 3.2 Forty‐one percent of phase I and II clinical trials for liver cancer in the US reported race/ethnicity data in the last 20 years

**FIGURE 2:**
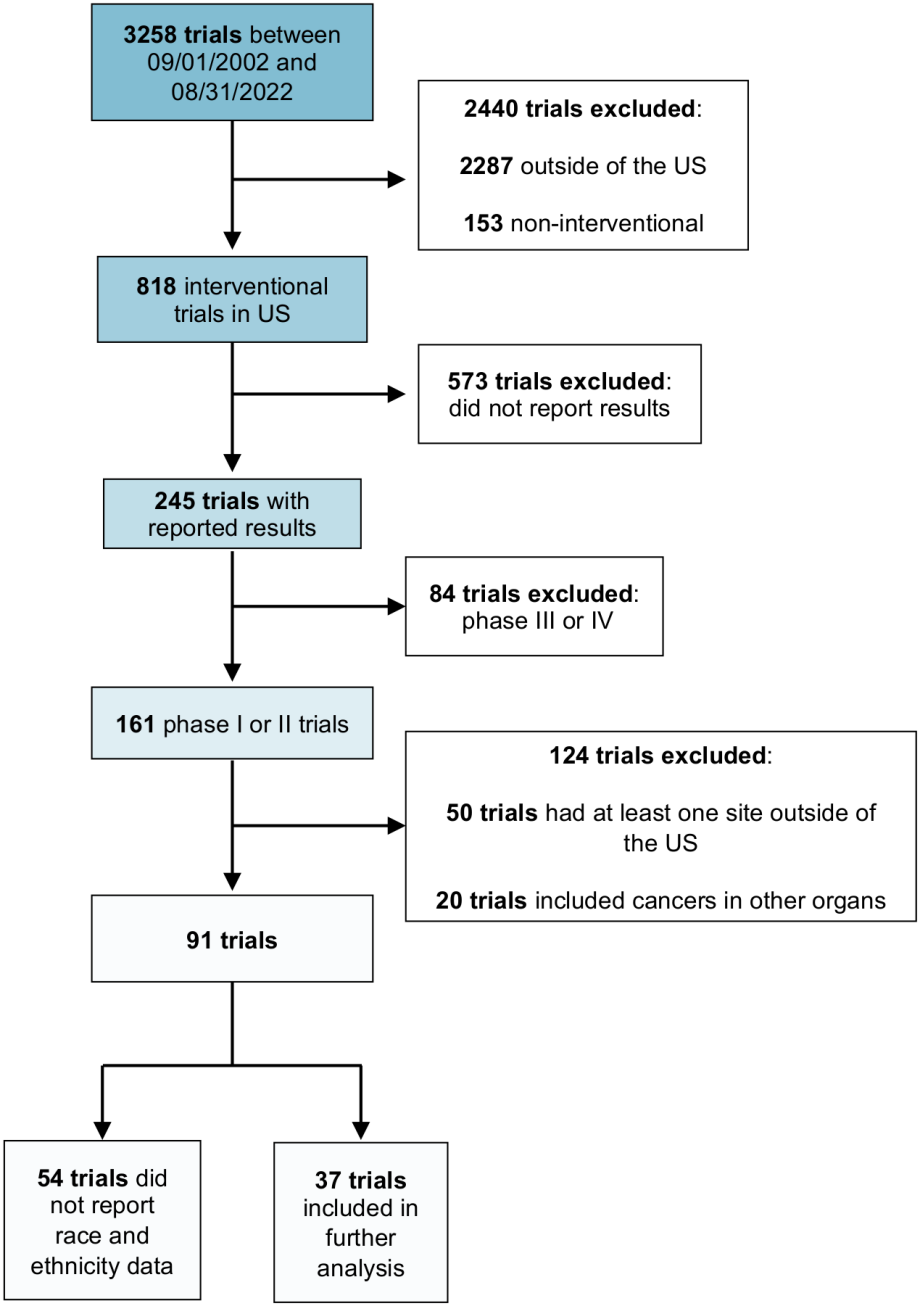
Consort diagram. A database search was performed in clinicaltrials.gov for phase I or II interventional liver cancer studies based only in the US with reported results from 09/2002 to 02/2023. Studies were excluded for: having sites outside of the US, being non‐interventional, not reporting results, being phase III or IV, including participants with cancer in other organs, and not reporting race and ethnicity data.

**FIGURE 3:**
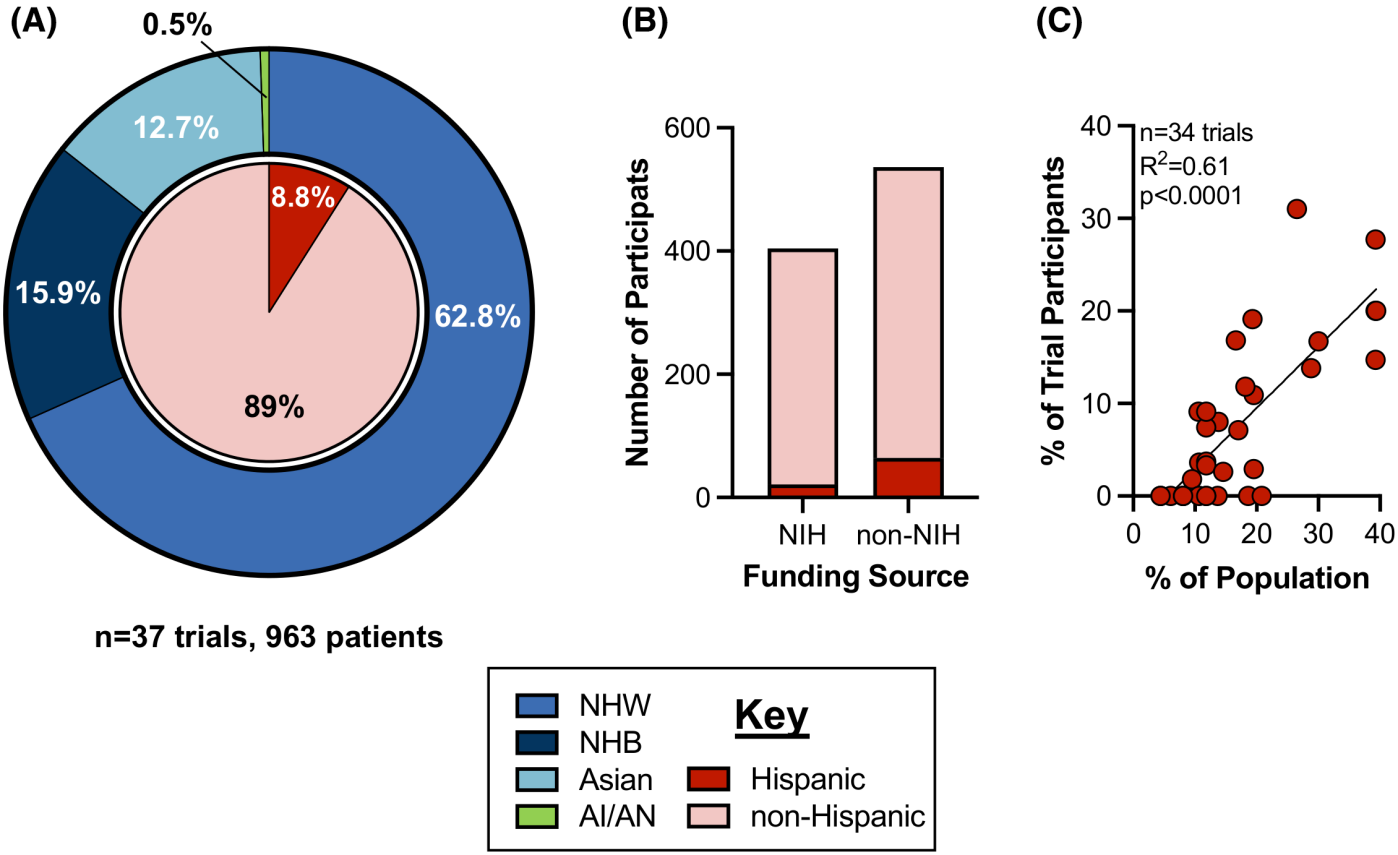
Demographics of patients enrolled in phase I and II clinical trials for liver cancer in the US (A) Enrollment data by race/ethnicity for *n* = 37 trials, 963 patients in US phase I and II clinical trials for cancer. (B) Number of Hispanic participants and non‐Hispanic participants in clinical trials that were NIH or non‐NIH‐funded. NIH‐funded *n* = 17 trials, 410 patients. 5.2% Hispanic, 94.8% non‐Hispanic. Non‐NIH‐funded *n* = 20 trials, 553 patients. 11.9% Hispanic, 88.1% non‐Hispanic (chi‐square analysis, *p* = 0.0004). (C) Linear regression analysis comparing proportion of Hispanic people in clinical trials vs proportion of Hispanic people in the population of the state(s) where such clinical trials were conducted. AI/AN, American Indian/Alaska Native individuals; NHW, non‐Hispanic white individuals, NHB, non‐Hispanic black individuals. Values for each pie charts do not add to 100%; the remaining percentage of participants is unknown or not reported.

A database search was performed in clinicaltrials.gov to identify phase I and II interventional liver cancer clinical trials based only in the US; 3258 trials were reported between September 1, 2002, and February 1, 2023. Trials were excluded that had recruitment sites outside of the US (*n* = 2287) or were non‐interventional (*n* = 153). Of the remaining 818 trials, trials were excluded because they did not report results to clinicaltrials.gov (*n* = 573), were phase III or IV (*n* = 84), had at least one site outside of the US (*n* = 50), and included participants with cancers in other organs (*n* = 20). Of the remaining 91 trials, 41% (*n* = 37 trials, 963 participants) reported race and ethnicity data (Figure 2). Among the 37 trials that reported race/ethnicity data, 62.8% were NHW people, 15.9% were NHB people, 12.7% were Asian people, 0.5% were AI/AN people, and 8.8% were Hispanic people (Figure 3A, Table 1).

**TABLE 1:** Clinical trials included in analysis. Percentage of total participants for each individual trial is displayed as well as sum of total participants for all *n* = 37 trials (*n* = 963 participants) and percentage of total participants for all trials for each race/ethnicity analyzed. AI/AN, American Indian/Alaska Native; NHB, non‐Hispanic black; NHW, non‐Hispanic white.

ct.gov identifier	Total Participants	Study start	Percent of Total					
			Hispanic	Non‐Hispanic	AI/AN	Asian	NHB	NHW
NCT00881751	95	03/09	16.8	83.2	1.1	13.7	15.8	63.2
NCT01853618	61	05/13	3.3	96.7	0.0	8.2	23.0	67.2
NCT01395030	57	08/11	1.8	98.2	0.0	50.9	3.5	22.8
NCT02821754	54	07/16	7.4	92.6	1.9	11.1	22.2	61.1
NCT01264705	47	01/11	27.7	72.3	0.0	6.4	36.2	29.8
NCT00906373	47	05/09	19.1	80.9	2.1	14.9	4.3	72.3
NCT00055692	46	02/03	10.9	89.1	0.0	30.4	4.3	54.3
NCT00627042	42	02/08	7.1	92.9	2.4	9.5	4.8	76.2
NCT01877187	39	04/13	2.6	97.4	0.0	0.0	59.0	25.6
NCT01900002	34	09/13	14.7	47.1	0.0	11.8	2.9	61.8
NCT00587067	34	06/03	2.9	91.2	0.0	5.9	0.0	88.2
NCT02658019	29	05/16	31.0	69.0	0.0	3.4	3.4	93.1
NCT01687673	29	10/12	13.8	86.2	0.0	34.5	3.4	62.1
NCT01642186	28	07/12	0.0	100.0	0.0	7.1	7.1	85.7
NCT00483405	28	10/06	3.6	96.4	0.0	0.0	32.1	60.7
NCT01306058	27	02/11	3.7	96.3	3.7	7.4	37.0	48.1
NCT00107536	26	10/05	0.0	96.2	0.0	3.8	15.4	76.9
NCT02273362	25	11/14	8.0	72.0	0.0	4.0	8.0	80.0
NCT02007954	24	02/14	0.0	95.8	0.0	4.2	29.2	62.5
NCT01488487	24	12/11	0.0	100.0	0.0	8.3	4.2	87.5
NCT01356628	23	05/11	0.0	95.7	0.0	30.4	17.4	52.2
NCT01099631	22	04/10	0.0	100.0	0.0	0.0	13.6	81.8
NCT02837029	17	07/16	11.8	88.2	0.0	0.0	5.9	82.4
NCT01766219	17	05/13	0.0	100.0	0.0	0.0	11.8	88.2
NCT01229111	14	10/10	0.0	100.0	0.0	7.1	14.3	78.6
NCT03111732	11	06/17	9.1	90.9	0.0	9.1	0.0	72.7
NCT02575339	11	07/16	9.1	90.9	0.0	0.0	0.0	90.9
NCT01375569	11	06/11	0.0	100.0	0.0	9.1	45.5	45.5
NCT03781960	7	07/19	0.0	100.0	0.0	28.6	14.3	42.9
NCT00787787	7	09/08	0.0	100.0	0.0	28.6	0.0	71.4
NCT01498952	6	01/12	16.7	83.3	0.0	16.7	16.7	50.0
NCT02695628	5	09/16	20.0	80.0	0.0	0.0	20.0	80.0
NCT02524119	5	04/16	20.0	80.0	0.0	0.0	20.0	60.0
NCT01754987	5	09/12	0.0	100.0	0.0	0.0	20.0	80.0
NCT02471313	3	06/15	33.3	66.7	0.0	0.0	33.3	66.7
NCT03433703	2	04/18	0.0	100.0	0.0	0.0	100.0	0.0
NCT03219372	1	09/18	0.0	100.0	0.0	0.0	100.0	0.0
sum of total participants	963		85	855	5	122	153	605
% of total participants for all trials			8.8	88.8	0.5	12.7	15.9	62.8

To better understand if there were any commonalities among clinical trials with a higher proportion of Hispanic participants, we further explored two factors: funding mechanism of the clinical trial and regional population characteristics. The NIH revitalization act established guidelines for inclusion of women and minorities in clinical research, and NIH‐funded studies must include plans for inclusion of racial/ethnic minorities within the application.16 Of the 37 trials in our analysis, 17 trials with 410 total patients were NIH‐funded and 20 trials with 553 total patients were non‐NIH‐funded. Hispanic people comprised 11.9% of participants in non‐NIH‐funded trials, significantly more than the 5.2% of participants in NIH‐funded studies (chi‐square, *p* < 0.0004) (Figure 3B).

People of Hispanic origin account for 19% of the US population, but the proportion of Hispanic people within individual states varies from 2% (in Maine) to 39.4% (in California).^20^ To determine if regional population characteristics had an impact on relative Hispanic enrollment in clinical trials, we utilized linear regression analysis. For multicenter studies, the percentage of the population was averaged for all states with clinical trial sites. Clinical trials conducted within states with a higher proportion of Hispanic residents had a higher enrollment of Hispanic patients (*n* = 34 trials with >5 total participants, R2 = 0.61, *p* < 0.0001) (Figure 3C). A similar correlation was seen with looking at only single‐center trials (*n* = 24 single‐center trials, R2 = 0.72, *p* < 0.0001) (Figure S1).

### 3.3 Hispanic patients are underrepresented in phase I and II clinical trials for liver cancer in the US

**FIGURE 4:**
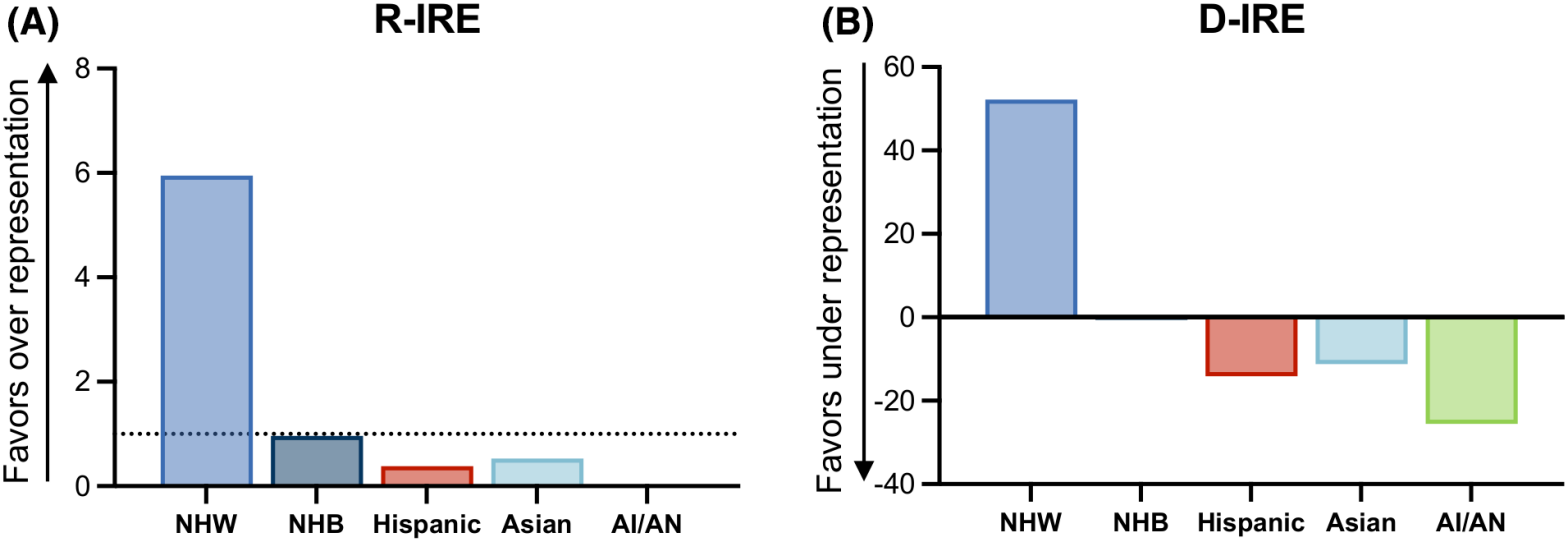
Underrepresentation of Hispanic individuals in clinical trials for liver cancer in the US. (A) difference in incident rate ratio (D‐IRE) by race and ethnicity and (B) the ratio of incidence by race and ethnicity (R‐IRE) for non‐Hispanic white (NHW) individuals, non‐Hispanic black (NHB) individuals, Hispanic individuals, Asian individuals, and American Indian/Alaska Native (AI/AN) individuals. D‐IREs below 1 and R‐IREs below 0 indicate underrepresentation in clinical trials based on relative incidence rate.

Despite representing less than 10% of patients enrolled in clinical trials for liver cancer in the US over the past 20 years, Hispanic people have accounted for 23% of all liver cancer cases in that same period of time. To understand how incidence rates compare with enrollment rates for each race/ethnicity, we calculated the difference in incidence by race and ethnicity (D‐IRE) and ratio of incidence rate by race and ethnicity (R‐IRE). D‐IREs below 1 and R‐IREs below 0 indicate underrepresentation in clinical trials based on relative incidence rate. Hispanic people had a D‐IRE of −14 and a R‐IRE 0.38, both indicating underrepresentation in clinical trials based on the relative incidence of liver cancer cases (Figure 4).

## 4 DISCUSSION AND CONCLUSIONS

Our findings suggest that there are significant disparities in the representation of racial/ethnic minority groups in liver cancer clinical trials in the US. The underrepresentation of Hispanic patients is particularly concerning, given their relatively high incidence of liver cancer. Hispanic people comprise the fastest‐growing subpopulation in the US and have the second‐highest incidence rate of liver cancer in the US (behind AI/AN people). They comprised only approximately 10% of patients in phase I or II clinical trials for liver cancer in the US in the past two decades, and we have not been able to observe any changes over time in the past 20 years. Two factors that were more common among trials with a higher enrollment of Hispanic people were being non‐NIH‐funded and having been conducted within states with more Hispanic residents. These findings may help develop strategies to increase enrollment rates of Hispanic patients. Additionally, less than half of the clinical trials in our analysis reported race/ethnicity data, perhaps limiting the ability to not only uncover differences in treatment outcomes for this population but to understand the true extent of the disparity as well. Future studies could focus on determining incentives or requirements to report such data.

Clinical trials are critically important for the development of novel therapies and can also provide possible treatment avenues for patients who do not respond to currently available therapies. However, only ~5% of adult cancer patients participate in clinical trials^21^ and future directions should further exploration the factors that contribute to this low participation rate. Some known barriers to participation such as access to care/treatment options, burden of travel, and financial burden may disproportionately affect minority groups and contribute to low participation in cancer trials in general and may also exacerbate the racial/ethnic disparities of those who do participate. Financial toxicity is important barrier to oncological care for both approved treatments and treatments accessed via clinical studies. Patients report a high economic burden associated with participation in clinical trials including both medical and non‐medical expenses, both of which are typically unexpected by participants. This financial toxicity is more common among lower‐income individuals and importantly, among non‐White and Hispanic patients.^19^ Possible incentives, which may help enhance enrollment rates for Hispanic patients include mandatory FDA guidelines for drug approval as well as mandatory rules by funding institutions and publishers.

This study is subject to some limitations. The results are based on a database search of clinical trials reported on clinicaltrials.gov and may not capture all trials. Additionally, the analysis is based on self‐reported race/ethnicity data and may not accurately reflect the true diversity of the trial participants. The SEER database that we used to acquire incidence data combines liver, intrahepatic bile duct, gallbladder, other biliary, pancreas, retroperitoneum, peritoneum, omentum, and mesentery, and other digestive organs into one category called “liver and intrahepatic bile duct cancer,” and however, the clinical trials used in this study enrolled patients with hepatocellular carcinoma only. This should be considered when interpreting the results comparing incidence data to enrollment data. It may be important for incidence databases to apply more granularity in their definitions of cancer sites. Finally, this study was limited to disparities in accurate representation of race/ethnicity in clinical trials and therefore did not look at other disparity parameters such as sex or socioeconomic status. Strength of the study are the long observation period (20 years) and its novelty and uniqueness as similar studies have not been conducted to the best of our knowledge.

Overall, this study highlights the need to further understand and address the underlying causes of racial/ethnic disparities in phase I and II clinical trials for liver cancer in the US to ensure that all patients have access to the same quality of care.

## AUTHOR CONTRIBUTIONS

**Cecilia Monge:** Conceptualization (equal); data curation (equal); formal analysis (equal); funding acquisition (equal); investigation (equal); methodology (equal); project administration (equal); resources (equal); software (equal); supervision (equal); validation (equal); visualization (equal); writing – original draft (equal); writing – review and editing (equal). **Tim F. Greten:** Conceptualization (equal); data curation (equal); formal analysis (equal); funding acquisition (lead); investigation (equal); methodology (equal); project administration (equal); resources (lead); software (equal); supervision (lead); validation (equal); visualization (equal); writing – original draft (equal); writing – review and editing (equal).

## FUNDING INFORMATION

TFG is supported by the Intramural Research Program of the NIH, NCI [ZIA BC 011343]. CM is a recipient of the Winn Diversity in Clinical Trials CDA.

## CONFLICT OF INTEREST STATEMENT

The authors do not have any disclosures or conflict of interests.

## Supporting information


Figure S1.

## Data Availability

The data generated for this study are available from the corresponding author upon reasonable request.
