# Supplementary figures and images for: Underrepresentation of Hispanics in clinical trials for liver cancer in the United States over the past 20 years
Source: Cancer Med. 2023 Dec 20;13(1):e6814. doi: 10.1002/cam4.6814 (PMC10807616; doi:10.1002/cam4.6814)

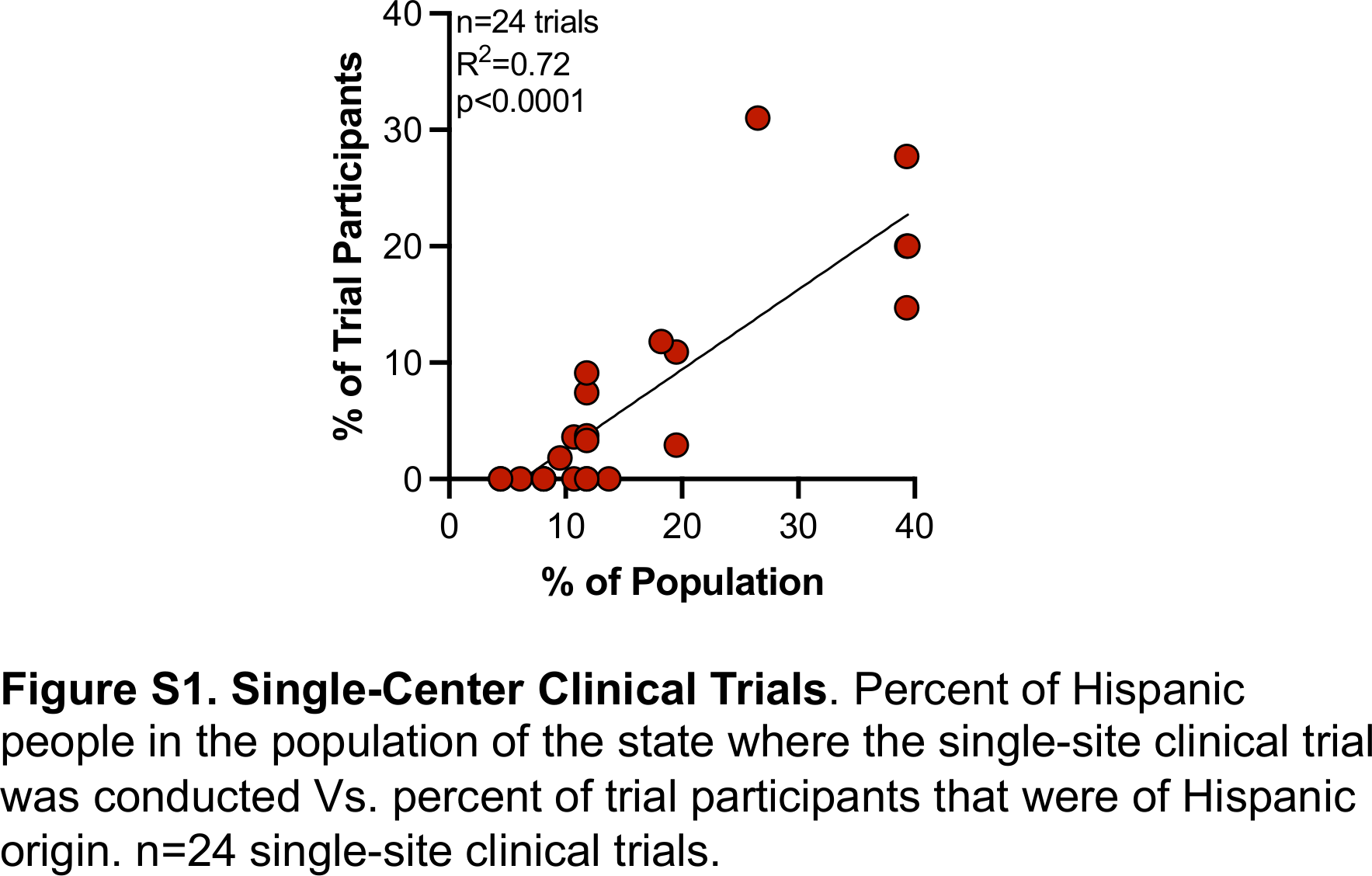

Supplement: Supplementary file 1 — Figure S1. [file CAM4-13-e6814-s001.tiff]
